# Shear Bond Strength and Bracket Base Morphology of New and Rebonded Orthodontic Ceramic Brackets

**DOI:** 10.3390/ma15051865

**Published:** 2022-03-02

**Authors:** Mihai Urichianu, Steven Makowka, David Covell, Stephen Warunek, Thikriat Al-Jewair

**Affiliations:** 1 Private Practice, San Antonio, TX 78212, USA; mihaiurichianu@gmail.com; 2 Dental Materials Research Laboratory, University at Buffalo School of Dental Medicine, Buffalo, NY 14214, USA; smakowka@buffalo.edu; 3 Department of Orthodontics, University at Buffalo School of Dental Medicine, Buffalo, NY 14214, USA; dacovell@buffalo.edu (D.C.J.); warunek@buffalo.edu (S.W.)

**Keywords:** ceramics, laser-etched bracket base, dental debonding

## Abstract

The objectives of this study were to (1) to evaluate the shear bond strength (SBS) of two ceramic brackets when new and when rebonded following various bracket base conditioning methods, and (2) to determine bond failure mode relative to bracket base morphology. 100 Symetri Clear^TM^ (SC) and 100 Radiance Plus^®^ (RP) ceramic brackets were bonded to bovine incisors and divided into five groups: one group served as controls and four had brackets rebonded following conditioning by: no surface treatment, sealant, sandblasting, and flame then steam. SBS, adhesive remnant index, and bracket base morphology were evaluated. SBS showed no statistical difference between new and rebonded with no surface treatment or sealant (SC brackets) and with sealant or flame and steam (RP brackets). When comparing SC to RP, SBS was higher with SC, no surface treatment, and sandblasted groups. All groups had varying amounts of adhesive left on the tooth, with the sandblasted group having the most. SEM analysis showed that sandblasting damaged the retention features of bracket bases. In conclusion, when rebonded, the SBS of SC brackets that had no surface treatment and both SC and RP brackets that had sealant showed no significant differences to new brackets. Sandblasting damaged the retention features of SC and RP bracket bases, resulting in low SBS.

## 1. Introduction

Ceramic brackets are commonly used in today’s orthodontic practice due to their esthetic properties, biocompatibility with oral tissues, comparable bond strengths to metal brackets, and resistance to thermal and chemical changes [1,2,3]. Despite their popularity, disadvantages have been noted for ceramic brackets including brittleness and enamel-adhesive bond failure during debonding [4,5,6]. Orthodontists often need to rebond brackets, for example to correct faulty initial positioning or to repair a bond failure between the bracket and tooth [7]. Rebonding of debonded brackets would save the cost of a replacement bracket, but decreased bond strength could be a potential tradeoff. For example, with chemically retentive ceramic brackets, Lew et al. [8] found that rebonded brackets have 30% less bond strength relative to new brackets. On the other hand, with mechanically retentive ceramic brackets, Harris et al. [9] found similar bond strengths between recycled and new brackets.

New generations of mechanically retentive ceramic brackets have incorporated innovative designs in their bracket bases to improve initial bond strength while simplifying the debonding process. Recently, two ceramic brackets have been introduced to the market that differ in their manufacturing process and design of the bracket base, with both aimed at providing a strong bond to enamel. The Symetri Clear^TM^ bracket (SC; Ormco, Brea, CA, USA) is made of a polycrystalline ceramic and the bracket base has a laser etched surface that is claimed to increase the reliability of bonding [10]. The Radiance Plus^®^ bracket (RP; American Orthodontics, Sheboygan, WI, USA) is made of monocrystalline ceramic and has a matte-textured base in the center of the bracket base and a smooth perimeter aimed at making debonding more predictable [11]. There are currently no studies comparing the shear bond strength (SBS) of newly bonded and rebonded SC and RP brackets.

The purpose of this study was to assess SBS, adhesive remnant index (ARI), and bracket base morphology of new SC and RP ceramic bracket bases, and when rebonded following different bracket base conditioning methods. Our null hypothesis was that there is no difference in SBS, ARI, and morphology between new and rebonded SC and RP brackets following different bracket base conditioning methods.

## 2. Materials and Methods

This ex-vivo randomized controlled trial involved the use of mandibular anterior SC (lot #101979927) and RP (lot #L31386) ceramic brackets, both with a prescription of 0.018-inch slot size, 0° torque, 0° angulation, and 0° rotation. Newly bonded brackets (N) were those that had not been previously bonded, whereas rebonded brackets (R) had been bonded and debonded prior to being bonded again. The brackets were bonded to bovine mandibular incisor teeth having sound facial enamel surfaces and no surface defects. Use of bovine teeth for research purposes had been reviewed by the University at Buffalo’s Institutional Animal Care and Use Committee (submission SIS07049N) and a determination was made that a review of the study protocol was not necessary.

A power analysis conducted based on detecting differences in SBS with a standard deviation of 7 MPa [12] and power of 90% found that at least 17 teeth would be needed in each group. A sample of 20 teeth per group was used to account for potential bracket fractures.

In addition, 100 of each bracket type were divided into five groups (*n* = 20 per group) where one group served as controls (N). The other 4 groups had brackets rebonded using different bracket base conditioning methods prior to rebonding, including: (1) no surface treatment (R-NST), (2) treated with sealant (R-S), (3) sandblasted (R-SB), or (4) treated with flame followed by steam (R-FS). The bovine teeth were disinfected by soaking in 1% chloramine-T solution for two weeks. The roots were then removed by sectioning approximately 1 mm apical to the cementoenamel junction, and the dental pulps were removed using cotton pliers. Facial surfaces were cleaned and polished for 10 s using non-fluoride pumice (Whip Mix, Louisville, KY, USA) on a rubber cup applicator in a low-speed hand piece.

Following manufacturers’ instructions, facial surfaces of each tooth were etched for 20 s with 35% phosphoric acid (Ultra Etch, Ultradent, South Jordan, UT, USA), then rinsed with water for 10 s, and air-dried for 5 s with oil-free compressed air. The facial surfaces were sealed with one coat of Assure^®^ Plus (Reliance Orthodontics, Itasca, IL, USA) using a brush pellet, and lightly dried with air. The sealant was then cured for 20 s using a VALO LED curing light (385–515 nm; Ultradent).

The brackets were bonded to the teeth by one operator (M.U.) using Transbond XT adhesive (3M, Monrovia, CA, USA). The adhesive was pressed against the bracket base using a spatula, and then the bracket was pressed onto the prepared enamel surface and excess was removed with an explorer. The adhesive was light-cured for 5 s on each side for a total of 20 s.

After all brackets were bonded, 20 teeth with SC and 20 teeth with RP brackets were randomly selected for the control groups and the remaining 160 bonded teeth allocated among the experimental groups. Brackets were debonded using a straight bracket removing plier (SC: REF#866-4020, Ormco; RP: REF#001-017405, American Orthodontics) according to manufacturers’ recommendations, using light pressure to produce a shear peel effect. Adhesive remnants were removed from enamel surfaces with a high-speed handpiece using a 12-fluted finishing carbide bur (REF# FG283-014; American Orthodontics) until the resin was no longer visible to the naked eye. A new bur was used after every 10 teeth.

The bonding protocol described earlier was then used to rebond brackets after the allocated base preparation was applied. Bracket bases in group R-NST received no additional treatment. Bracket bases in group R-S were sealed with one coat of Assure^®^ Plus using a brush pellet, lightly dried with air, and cured for 20 s using the VALO LED curing light. The bracket bases in group R-SB were sandblasted with a Microetcher II (Great Lakes Dental Technologies, Tonawanda, NY, USA) using 50 µm aluminum oxide particles at 65 psi for 20–30 s until there was no resin remaining to the naked eye. They were then rinsed with water for 10 s and dried with compressed air. The bracket bases in group R-FS were treated with a flame and steam protocol developed by the authors of this study. Flame treatment was performed using the non-luminous zone of a butane lab torch (GB 2001 Micro Torch, Patterson Dental, Saint Paul, MN, USA) with 3 sweeping motions, each sweep lasting approximately 1 s, followed by a jet of steam (Triton SLA, BEGO Inc., Lincoln, RI, USA) held 1” away from the bracket base for 3 s. The teeth in all five groups were then embedded in 1” diameter cylindrical acrylic resin blocks for SBS evaluation.

SBS was tested using an UltraTester (Ultradent) with a crosshead speed of 1 mm/min. The straight blunt blade was directed between the bracket wing and base and parallel to the bracket slot as described by Halpern and Rouleau [13]. The SBS was recorded in Newtons and in MPa, calculated as the ratio of force to bracket base surface area in mm^2^. To assess the clinical relevance of the SBS values obtained, a range found in the literature of 5.9–7.8 MPa [14,15] to 8–16 MPa [12,16,17] was considered, and 5.9–16 MPa was considered the clinically acceptable range.

The ARI was determined according to Artun and Bergland [18], assessing the amount of adhesive remaining on the enamel vs. bracket. Scores from 0–3 were assessed as follows: 0: no adhesive left on the tooth surface; 1: <50% of the adhesive left on the tooth surface; 2: >50% adhesive left on the tooth; and 3: all adhesive left on the tooth surface with a distinct impression of the bracket base. Three brackets from each group, the lowest, median, and highest SBS samples, were evaluated with a stereomicroscope (Dino-Lite Edge, Dino-Lite, Taiwan) at a constant magnification that captured the entire bracket base (22.7×). Digital images were recorded using DinoCapture 2.0 Microscope Imaging Software (Dino-Lite, Torrance, CA, USA). 

Bracket base morphology was compared pre- and post- bonding by scanning electron microscopy (SEM; Hitachi SU70 FESEM, Tokyo, Japan). Ten samples were selected, one representative bracket that had a median SBS from each group. To detect any traces of enamel, residual composite and ceramic, surfaces were characterized by a combination of secondary electrons and Energy Dispersive Spectroscopy (EDS) elemental analysis (IXRF X-ray analyzer, Austin, TX, USA).

Data were analyzed using SPSS version 24 for Windows. To evaluate intra-examiner reliability of the ARI, 20 randomly selected brackets, two from each group, were reassessed by the same examiner two weeks later and intra-class correlation coefficients (ICC) were calculated. All data sets were assessed for normality using the Kolmogorov–Smirnov test. The test indicated violations of normality for three of the 10 groups; therefore, the Kruskal–Wallis test was used for pairwise SBS comparisons among the five groups within the two bracket types. Mann–Whitney’s test was used to compare bond strengths between SC and RP groups. The Chi-Square test was used to compare ARI results among the 10 groups. Due to the number of comparisons, to decrease the chance of Type I errors, the significance level was adjusted by a Bonferroni correction from *p* < 0.05 to *p* < 0.01.

## 3. Results

### 3.1. Shear Bond Strength

During SBS testing, 8 SC (8%) and 1 RP brackets (1%) fractured and were excluded from further analysis. Table 1 shows the median SBS for the two bracket types. For the SC brackets, SBS from highest to lowest were N, R-NST, R-S, R-FS and R-SB, with a median SBS ranging from 3.62 ± 1.88 to 8.31 ± 3.84 MPa. Median SBS were within the clinically acceptable range for N, R-NST, and R-S groups. For the RP brackets, the order from highest to lowest was N, R-S, R-FS, R-NST, and R-SB, with a median SBS ranging from 1.08 ± 0.85 to 8.35 ± 3.87 MPa. Median SBS were within the clinically acceptable range for N and R-S groups.

Table 2 shows pairwise comparisons of the SBS among the five groups by bracket type. Within the SC brackets, significant differences were found between N vs. R-SB groups (*p* < 0.001), N vs. R-FS (*p* < 0.001), and R-NST vs. R-SB (*p* = 0.003). Within the RP brackets, significant differences were found between N vs. R-NST (*p* = 0.01), N vs. R-SB groups (*p* < 0.001), R-NST vs. R-SB (*p* = 0.001), R-S vs. R-SB groups (*p* < 0.001), and R-SB vs. R-FS groups (*p* < 0.001).

Table 3 shows pairwise comparisons of SBS between SC and RP groups. Significant differences between bracket types were found for the R-NST groups (*p* = 0.009) and the R-SB groups (*p* < 0.001).

### 3.2. Adhesive Remnant Index

Intra-examiner reliability assessed by repeating the ARI had an ICC of 1.00, indicating excellent reproducibility. Table 4 shows the distribution of ARI values within each bracket type. There were no ARI values of zero. For ARI score of 1, the R-NST group had the most values (SC: 22.2%; RP: 21.1%), for a score of 2, the N group had the highest number of values (SC: 90%; RP: 95%), and, for a score of 3, the R-SB group had the most values (SC: 73.7%; RP: 100%). When ARI values were compared using the Chi-Square test, no significant differences were found.

**Figure 1:**
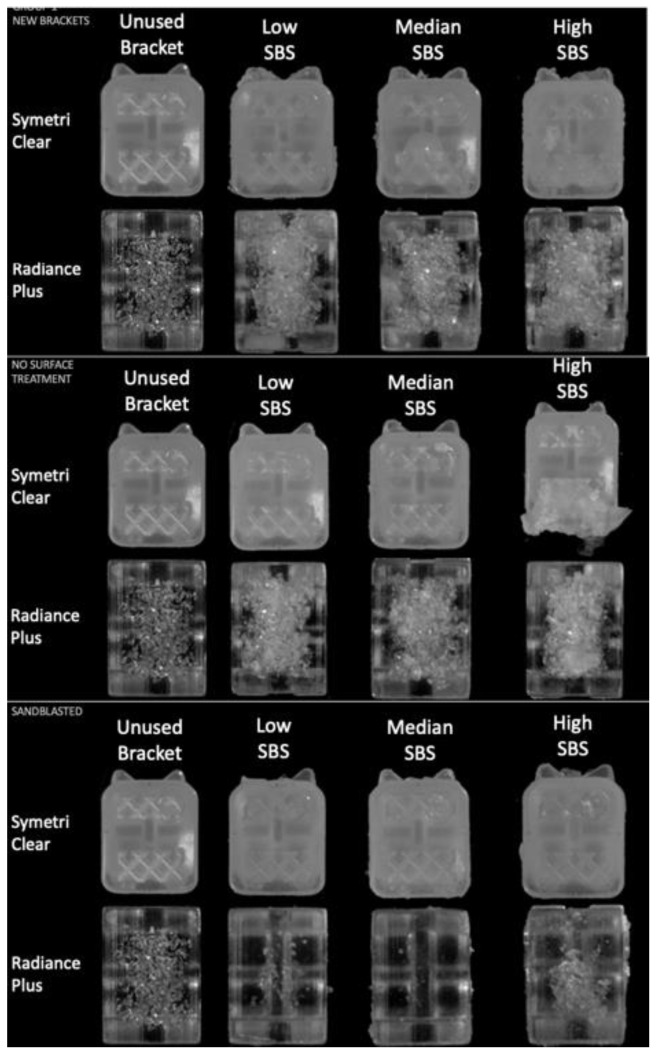
Stereomicroscope Images of low/median/high shear bond strength for new (N), rebonded and no-surface treatment (R-NST), and rebonded and sandblasted (R-SB) representative samples.

As shown in Figure 1, when brackets were inspected for adhesive remnants using the stereomicroscope, all the debonded groups showed bracket bases with adhesive attached to the retention features. No pattern was observed regarding the amount of remnant adhesive in the representative samples having low, medium and high SBS, except with the RP bracket in the R-SB group. With the latter, there was no evidence of adhesive in the low and medium SBS samples, whereas composite remnants were observed in the high SBS sample.

### 3.3. Bracket Base Morphology

**Figure 2:**
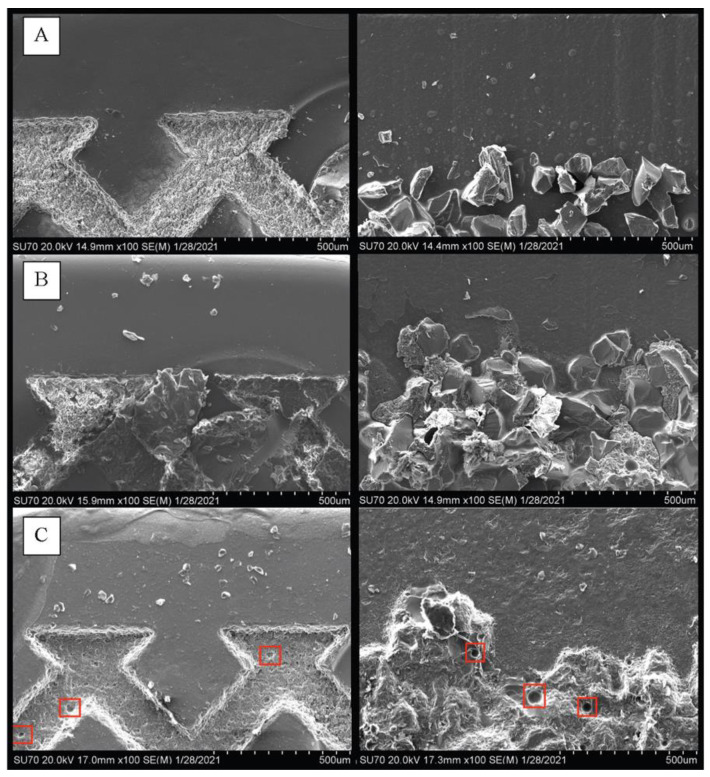
(**A**) SEM of unused SC (left) brackets showing the laser etched retentive feature, and RP (right) brackets showing the matt-textured, crystalline retentive feature. (100× initial magnification); (**B**) New SC (left) and RP (right) brackets following debonding. With SC, adhesive remnants partially cover the laser etched retention feature. With RP, gaps between the crystalline structures contain adhesive remnants. For both bracket types, minimal remnants of adhesive were present on the smooth surface. (100× initial magnification); (**C**) Rebonded and sandblasted SC (left) and RP (right) brackets (R-SB). The red squares show some of the damage done by the sandblasting particles. Note that, while the laser etched pattern appears intact in the SC brackets, much of the crystalline structure has been removed with the RP brackets (100× initial magnification).

Upon inspection of the bracket base morphology under the stereomicroscope and SEM (Figure 2), the bases of unused (before bonding) SC brackets showed a smooth base with two rows of laser-etched, crisscrossed grooves. The unused RP bracket bases showed a smooth perimeter area and irregular, crystalline structures protruding from the central region. To further investigate the crystalline structures on the RP bracket bases, EDS determined that there were aluminum oxide particles along with surface droplets composed of silicon, calcium, and sodium.

Following debonding, when the retentive and smooth areas of the N brackets were examined by SEM (Figure 2), adhesive remnants were found to be associated with the retentive features, partially occupying the laser-etched pattern of the SC brackets, and filling gaps between the crystalline structures of the RP brackets. The smooth surfaces had almost no remnants. With the R-SB groups, it was found that the retention features of the brackets had been damaged by sandblasting treatment, particularly the crystalline structure of the RP brackets.

## 4. Discussion

This study compared the SBS of two newly marketed ceramic brackets with differing adhesive retention features. The brackets were assessed when new and after rebonding following four different bracket base treatments. With the SC brackets, the median SBS of three groups fell into the clinically acceptable range (5.9–16 MPa): N (8.31 ± 3.84 MPa), R-NST (6.84 ± 4.36 MPa) and R-S (6.47 ± 3.14 MPa). With the RP brackets, the median SBS of two groups fell into the clinically acceptable range: N (8.35 ± 3.87 MPa) and R-S (6.24 ± 3.57 MPa). Consistent with previous studies [12,17,19,20], the N brackets had the highest overall median SBS, with no significant difference between the two types. The R-NST group with the SC bracket had the second-highest median SBS (6.84 ± 4.36 MPa), where the value was within the clinically acceptable bond strength range, whereas, with the RP bracket, the R-NST group had the fourth highest median SBS (4.12 ± 3.83 MPa), where the value was below the acceptable range. Comparison of the R-NST group by bracket type showed a statistically significant difference (*p* = 0.009). An explanation for the difference likely relates to the extent that the brackets’ initial mechanical retention systems remained functional following initial debonding. SEM imaging showed that, following debonding, the SC bracket had areas where the laser-etched retention pattern appeared intact, whereas the RP brackets had composite remnants filling the areas between the crystals of the retentive feature. Our results differ from those of Ballard et al. [21], who analyzed the tensile bond strength of SC brackets after initial bonding and, after rebonding for a second- and third time, with and without use of a bracket base conditioner. They found rebonding brackets without use of a conditioner resulted in a significant reduction in bond strength. The differences between the two studies could be attributed to the bond strength test utilized, tensile vs. shear. Many previous studies [9,12,17,22,23] have used SBS; therefore, the current study used SBS in order to compare our results more directly to those of other studies.

The R-S group had the third-highest median bond strength (6.47 ± 3.14 MPa) for the SC brackets and the second-highest strength (6.24 ± 3.57 MPa) for the RP brackets. These results suggest that clinically acceptable bond strengths can be achieved with either bracket type when rebonded after conditioning the bracket base with sealant, and there was no significant difference in SBS between the two groups. For the SC bracket, current results are consistent with those of Ballard et al. [21] in the study mentioned above, where they found no significant differences in bond strength between initially bonded and rebonded SC brackets when the bracket base was conditioned with sealant.

The R-SB group presented the lowest median bond strengths for both SC (3.62 ± 1.88 MPa) and RP (1.08 ± 0.85 MPa) brackets, and the difference between the two brackets was statistically significant (*p* < 0.001). While sandblasting bracket bases is the gold standard before rebonding metal brackets, current results weigh against this treatment method with ceramic brackets. Current results are consistent with those obtained by others who tested rebonded polycrystalline ceramic brackets following sandblasting. Chung et al. [12], assessing Clarity brackets (3M, Monrovia, CA, USA), and Toroglu et al. [3], assessing Inspire brackets (Ormco, Brea, CA, USA), found low bond strengths, 2.97 ± 2.29 MPa and 4.5 ± 2.1 MPa, respectively, strengths that are below clinically acceptable values. The SEM analysis in our study showed that the 50 µm sandblasting particles damaged the laser etched features of the SC brackets and removed much of the aluminum oxide crystalline structure of the RP brackets. Montero et al. [24] compared the shear bond strength of both metal and ceramic brackets after being sandblasted with different sizes of aluminum oxide particles (25, 50, and 110 µm) and found no significant difference among groups. Given these findings, it is likely that using different sizes of aluminum oxide particles would not have altered the results of the current study.

The R-FS group scored fourth-best for the SC bracket (3.72 ± 4.39 MPa) and third-best for the RP brackets (4.57 ± 2.28 MPa). The difference between the two was not significant and neither had bond strengths that would be clinically acceptable, although the R-FS for the RP bracket did not differ statistically from the N RP brackets. The rationale for choosing this treatment was that the flame would burn off much of the methacrylate-based adhesive and contaminants that remained upon debonding, and then the steam would remove the residual bonding material, leaving a clean, undamaged bracket base. SBS results demonstrated that this method was relatively ineffective for SC brackets but had less adverse effects for the RP brackets. Potentially, a much higher temperature than the non-luminous zone of the butane lab torch may be needed to break down the resin remnants. While there is no previous research on the bond strength of ceramic brackets using flame followed by steam treatment prior to rebonding, two studies [19,20] have tested the effect of heat treatment on bond strength. Silva et al. [19] tested the SBS of rebonded ceramic brackets after they were heat treated with a hot air dryer for 60 s. The results were consistent with this study for the SC bracket in that the SBS was significantly lower than that of new brackets. Consistent with our results for the RP bracket, Mirhashemi et al. [20] obtained SBS for a flame-treated group where the values trended lower, although not significantly, when compared to that of new brackets. A possible reason why results of Mirhashemi et al. [20] are not consistent with our findings for the SC brackets (N vs. R-FS) is the difference in rebonding protocol where the Mirhashemi et al. [20] study included cleaning the base in an ultrasonic bath for five minutes to remove impurities, versus use of steam in the current study. Overall, the findings for the R-SB and R-FS groups lead to the rejection of the null hypothesis that there is no difference in SBS between new ceramic brackets and rebonded ones with or without surface conditioning.

The ARI assessment indicated no significant differences between the adhesive-bracket interface failures among the treatment groups for the SC and RP bracket types. Because the ARI results indicated no difference between the new and rebonded ceramic brackets, this portion of the null hypothesis was not rejected. Similar to findings in other studies [12,20,25], the ARI analysis indicated for most groups a combination of adhesive and cohesive failure occurring at the resin–bracket interface (ARI 1, 2). The finding of no ARI = 0 scores can be considered advantageous as enamel fractures may be more likely to occur with this pattern of debonding [18]. Brackets in the N groups also had no ARI = 3 scores, indicating that all of the adhesive was left on the tooth surface, whereas the rebonded groups had such scores ranging from 5.9 to 100%. This can be attributed to new brackets having adequate retention features, allowing the resin to mechanically adhere to the bracket bases, whereas the retentive features had diminished effectiveness in the rebonded groups, a finding that was also confirmed by direct observations of bracket bases in low, medium and high SBS samples. When observed with microscopy, brackets with higher SBS values had more adhesive retained on the retentive features relative to those with lower SBS values. The R-SB groups for both SC and RP brackets presented with the highest number of ARI = 3 values. This can be explained by the damage imparted on the retention features by the sandblasting. The results of this study indicate against sandblasting SC and RP ceramic brackets before rebonding. On the other hand, the recent study of Ballard et al. [21] suggests that, if sealant is used after sandblasting, adequate bond strengths can be achieved with the SC bracket.

An incidental finding in the current study was that, during SBS testing, 8% of the SC brackets fractured, compared to 1% of the RP brackets. SC brackets are polycrystalline, made in a mold from multiple fused aluminum oxide particles, while RP brackets are monocrystalline, milled from a block of aluminum oxide [26]. The main advantage of polycrystalline over monocrystalline brackets is that they are less expensive to manufacture and can be produced rapidly in large quantities [26]. However, relative to monocrystalline brackets, polycrystalline brackets are more prone to structural imperfections and impurities that can cause propagation of cracks under stress [26]. Da Rocha et al. [17] in vitro measured the SBS of three different types of polycrystalline ceramic brackets and found that 38% of the brackets fractured when they were debonded. Our results showing increased fracturing of polycrystalline relative to monocrystalline brackets are consistent with these findings.

Potential limitations of the current study were that chewing simulation by cyclic loading was not performed. Imani et al. [27], however, concluded that thermocycling (500 cycles) and cyclic loading (10,000 cycles) did not affect SBS of orthodontic brackets. Future studies, however, must still consider thermocycling and cyclic loading in their methodologies. In addition, treatment with sandblasting or flame and steam followed by sealant was not investigated and could be the focus of a future investigation. For example, a study using a polycrystalline bracket bonded to extracted human premolars found that rebonding and sandblasting followed by applying sealant resulted in clinically acceptable results (7.65 ± 5.62 MPa) [12]. Finally, with the advances in 3D printing and CAD technologies, new generations of 3D printed ceramic brackets have become available. Yang et al. [28] recently compared the mechanical properties of custom printed ceramic brackets to commercially available mono- and polycrystalline brackets and found no significant differences in SBS between the groups. Future studies assessing the SBS of bonded and rebonded custom printed ceramic brackets after various conditioning methods are warranted.

## 5. Conclusions

For SC brackets, N, R-NST, and R-S yielded SBS results that showed no statistical differences and had acceptable clinical bond strengths. Therefore, although sealant can be used, no surface treatment of debonded brackets is an option when rebonding. With the RP groups, N, R-S and R-FS yielded SBS results that showed no statistical differences. However, only N and R-S had clinically acceptable bond strengths. Therefore, sealant should be used when rebonding. Sandblasting damaged the retention features of the SC brackets and especially that of the RP brackets. There was no difference in ARI findings between SC and RP among the five experimental groups.

## Figures and Tables

**Table 1:** Shear bond strengths (MPa) of newly bonded and rebonded brackets.

Bracket Type *	Group ^†^	Median	IQR	25th Percentile	75th Percentile	Mean	SD
SC	N	8.31	3.84	6.67	10.51	9.15	3.39
	R-NST	6.84	4.36	4.92	9.29	7.14	3.00
	R-S	6.47	3.14	4.35	7.49	6.28	2.24
	R-SB ^#^	3.62	1.88	2.93	4.81	3.83	1.38
	R-FS ^#^	3.72	4.39	2.16	6.55	4.36	2.51
RP	N	8.35	3.87	6.41	10.27	8.45	2.97
	R-NST ^#^	4.12	3.83	2.65	6.48	4.67	2.23
	R-S	6.24	3.57	4.23	7.81	6.42	2.29
	R-SB ^#^	1.08	0.85	0.79	1.63	1.27	0.68
	R-FS ^#^	4.57	2.28	3.29	5.57	4.74	1.69

* Bracket types: Symetri Clear™ (SC); Radiance Plus^®^ (RP); ^†^ Group categories: New brackets (N); Rebonded brackets—with no surface treatment (R-NST); treated with sealant (R-S); sandblasted (R-SB); and treated with flame and steam (R-FS). ^#^ Median value outside clinically acceptable SBS range (5.9–16 MPa).

**Table 2:** Pairwise statistical comparisons of shear bond strengths within bracket groups.

Group		N	R-NST	R-S	R-SB	R-FS
**N**	*SC*	-	0.974	0.246	<0.001 *	<0.001 *
	*RP*	-	0.010 *	0.889	<0.001 *	0.013
**R-NST**	*SC*	0.974	-	1.000	0.003 *	0.043
	*RP*	0.010 *	-	0.722	0.001 *	1.000
**R-S**	*SC*	0.246	1.000	-	0.024	0.221
	*RP*	0.889	0.722	-	<0.001 *	1.000
**R-SB**	*SC*	<0.001 *	0.003 *	0.024	-	1.000
	*RP*	<0.001 *	0.001 *	<0.001 *	-	<0.001 *
**R-FS**	*SC*	<0.001 *	0.043	0.221	1.000	-
	*RP*	0.013	1.000	1.000	<0.001 *	-

* Kruskal–Wallis test, *p* ≤ 0.01.

**Table 3:** Shear bond strength comparisons between Symetri Clear™ and Radiance Plus^®^ groups.

SC vs. RP	*p*-Value
N	0.698
R-NST	0.009 *
R-S	0.988
R-SB	<0.001 *
R-FS	0.244

* Independent samples Mann–Whitney Test; *p* ≤ 0.01.

**Table 4:** Adhesive Remnant Index (ARI) frequencies *.

Bracket Type	Group	ARI = 0	ARI = 1	ARI = 2	ARI = 3	Total
		N (%)	N (%)	N (%)	N (%)	N (%)
SC	N	0 (0.0)	2 (10.0)	18 (90.0)	0 (0.0)	20 (100.0)
	R-NST	0 (0.0)	4 (22.2)	11 (61.1)	3 (16.7)	18 (100.0)
	R-S	0 (0.0)	2 (11.1)	14 (77.8)	2 (11.1)	18 (100.0)
	R-SB	0 (0.0)	0 (0.0)	5 (26.3)	14 (73.7)	19 (100.0)
	R-FS	0 (0.0)	3 (17.6)	13 (76.5)	1 (5.9)	17 (100.0)
RP	N	0 (0.0)	1 (5.0)	19 (95.0)	0 (0.0)	20 (100.0)
	R-NST	0 (0.0)	4 (21.1)	13 (68.4)	2 (10.5)	19 (100.0)
	R-S	0 (0.0)	3 (15.0)	14 (70.0)	3 (15.0)	20 (100.0)
	R-SB	0 (0.0)	0 (0.0)	0 (0.0)	20 (100.0)	20 (100.0)
	R-FS	0 (0.0)	0 (0.0)	15 (75.0)	5 (25.0)	20 (100.0)
	**Total**	**0 (0.0)**	**19 (9.9)**	**122 (63.9)**	**50 (26.2)**	**191 (100.0)**

* ARI Scoring: 0: no adhesive left on the tooth surface; 1: ≤50% of the adhesive left on the tooth surface; 2: >50% adhesive left on the tooth; and 3: all adhesive left on the tooth surface, with a distinct impression of the bracket base.

## Data Availability

Not applicable.
